# Responses to Drought Stress in Poplar: What Do We Know and What Can We Learn?

**DOI:** 10.3390/life13020533

**Published:** 2023-02-15

**Authors:** Laura Rosso, Simone Cantamessa, Sara Bergante, Chiara Biselli, Agostino Fricano, Pier Mario Chiarabaglio, Massimo Gennaro, Giuseppe Nervo, Francesca Secchi, Andrea Carra

**Affiliations:** 1 Council for Agricultural Research and Economics, Research Centre for Forestry and Wood, Strada Frassineto 35, 15033 Casale Monferrato, Italy; 2 Council for Agricultural Research and Economics, Research Centre for Viticulture and Enology, Viale Santa Margherita 80, 52100 Arezzo, Italy; 3 Council for Agricultural Research and Economics, Research Centre for Genomics and Bioinformatics, Via San Protaso 302, 29017 Fiorenzuola d’Arda, Italy; 4 Department of Agricultural, Forest and Food Sciences, University of Turin, Largo Paolo Braccini 2, 10095 Grugliasco, Italy

**Keywords:** water, trees, stress, tolerance, stomata, xylem embolism, drought-responsive genes

## Abstract

Poplar (*Populus* spp.) is a high-value crop for wood and biomass production and a model organism for tree physiology and genomics. The early release, in 2006, of the complete genome sequence of *P. trichocarpa* was followed by a wealth of studies that significantly enriched our knowledge of complex pathways inherent to woody plants, such as lignin biosynthesis and secondary cell wall deposition. Recently, in the attempt to cope with the challenges posed by ongoing climate change, fundamental studies and breeding programs with poplar have gradually shifted their focus to address the responses to abiotic stresses, particularly drought. Taking advantage from a set of modern genomic and phenotyping tools, these studies are now shedding light on important processes, including embolism formation (the entry and expansion of air bubbles in the xylem) and repair, the impact of drought stress on biomass yield and quality, and the long-term effects of drought events. In this review, we summarize the status of the research on the molecular bases of the responses to drought in poplar. We highlight how this knowledge can be exploited to select more tolerant genotypes and how it can be translated to other tree species to improve our understanding of forest dynamics under rapidly changing environmental conditions.

## 1. Introduction

Current climate change is characterized by global warming and a shift in rainfall patterns, resulting in increased frequency of heavy rain episodes separated by long dry spells over large geographic areas [1]. In these conditions, abiotic stresses, especially drought, are predicted to become more significant, posing new challenges to ecosystems and agricultural activities [1]. The impact of drought stress can be particularly severe on forest trees due to two intrinsic vulnerabilities, related to their size, which necessitates a complex vascular water-transport system from soil to canopy, and their long generation time, which results in slow genetic adaptation [2,3,4]. Considering that forests cover about one third of terrestrial land [5], host more than half of terrestrial biodiversity, sequester and store large quantities of CO_2_, and are powerful drivers of the Earth’s water cycle, forest dieback due to drought is regarded with increasing concern by the scientific community [6,7]. The impact of climate change on forest dynamics has been addressed in several recent reviews [2,3,4,8].

Among trees that have been domesticated and are grown in agricultural lands, poplar (*Populus* spp.) is one of the higher-value crops for woody biomass production. Global poplar plantations cover approximately 31.4 million hectares. The largest cultivated areas are found in Canada and China, followed by Europe where France and Italy are the main producers. In Italy, plantations are in the Po’ valley cover about 43,400 hectares [9]. Furthermore, poplar is a model organism for tree physiology and genomics thanks to its small genome size, fast growth rate and ease of transformation [10]. Following the early release of the complete genome sequence of *P. trichocarpa* [11], the number of published studies on poplar genomics has been growing exponentially. These studies offer us a chance to understand how trees respond to environmental challenges, paving the path for the development of effective strategies for sustainable forest management and breeding programs. Intensive genetic improvement programs were implemented for poplar from the early years of the 20th century, with three main objectives: yield, pest and pathogen resistance and wood quality [12]. Nowadays, it is necessary to adjust these objectives to increase the focus on abiotic stress tolerance.

Drought affects plants in several ways. Osmotic stress due to solute concentration damages cellular components and causes loss of turgor that limits cell wall expansion and organ growth. The closure of stomatal pores, which is a common response to drought, reduce CO_2_ uptake and fixation leading carbon imbalance and depletion of carbohydrate reserves. Stomatal closure also reduces transpiration, limiting leaf evaporative cooling with consequent heat-stress impact on metabolic processes, primarily photosynthesis. The accumulation of reactive oxygen species (ROS), which is another common effect of drought stress, damages proteins and membranes. Furthermore, decreased soil water potential exposes the xylem transport system to risk of cavitation and embolism formation that can lead to hydraulic failure and death of branches or even of the whole plant [13,14,15,16].

**Figure 1:**
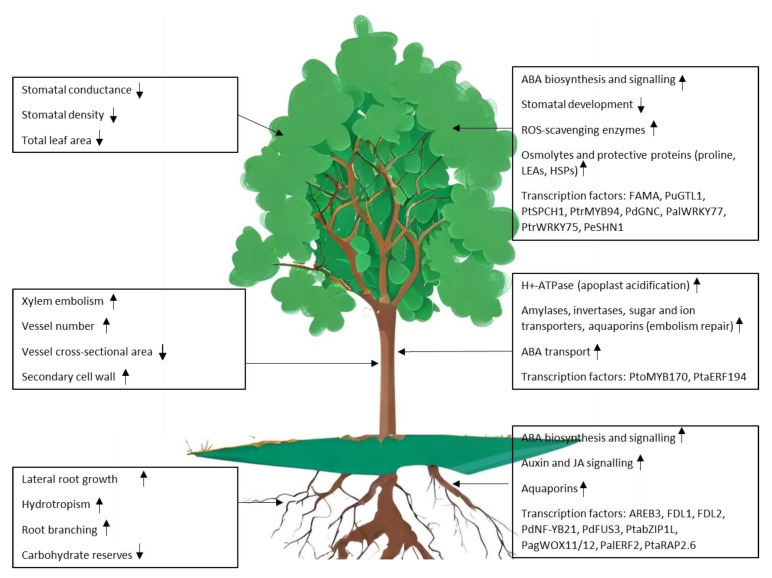
Responses to drought stress in poplar: physiological and morphological changes in leaves, stem and roots (left panels) are controlled by the activation of signalling pathways and drought-responsive genes (right panels).

Poplar adopts two main strategies to cope with water deficit: drought avoidance and drought tolerance [17]. Avoidance mechanisms include the control of transpiration through the regulation of stomatal conductance, deposition of cuticular waxes to limit non-stomatal transpiration, increased root growth, reduced leaf area and leaf shedding [7]. Drought avoidance strategies are particularly effective in trees, as they can generally rely on a high degree of phenotypic plasticity to adjust their morphological traits [18,19,20]. Tolerance mechanisms are aimed at maintaining biological functions under stress conditions; these encompass the accumulation of osmolytes (glycine betaine, proline, sugars) that help maintain water fluxes and cell turgor [21,22], the synthesis of molecules that exert a protective action on proteins and membranes as late embryogenesis abundant (LEA) proteins or proline, the expression of genes encoding ROS scavenging enzymes and aquaporins, channel proteins that contribute to water fluxes across membranes [23,24]. The deployment of avoidance and tolerance mechanisms is energetically costly and involves a trade-off between stress resilience and growth (Figure 1).

## 2. Drought Response in Poplar Is a Complex Trait

Poplar genotypes differ widely in terms of how they prioritize the trade-off between drought resilience and growth; in general, the most productive under non-limiting water conditions display the greatest reduction in yield under drought conditions [25,26]. This variability has been found in and between poplar species and is correlated with water availability in their natural habitat [27]. Recently, genetic mapping and genome-wide association studies (GWAS) have started to explore this partially untapped diversity to unravel the genetic architecture of drought response in poplar [28,29,30]. Using contrasting poplar species, an interspecific F1 population of 144 individuals derived from a crossing between *P. deltoides* and *P. simonii* was used to analyse five drought-related traits at the seedling stage. This analysis led to the identification of 63 quantitative trait loci (QTLs) that co-segregated with traits associated with drought tolerance [28]. Interestingly, the functional annotation of genes identified within the QTLs indicated a broad variety of gene functions. Furthermore, using two parental genotypes of *P. deltoides* and *P. trichocarpa* showing contrasting physiological traits, 54 QTLs specific for drought response were mapped from an F2 population phenotyped under controlled conditions. Several of these QTLs traits showed overlapping positions [29]. Low osmotic potential at full turgor in leaves has been proposed and employed as a proxy for measuring the degree of dehydration in poplar. The use of an F2 population derived from a *P. trichocarpa* × *P. deltoides* cross was extensively phenotyped for low osmotic potential at full turgor in presence of limiting and non-limiting water conditions. The screening allowed researchers to identify seven significant QTLs accounting for 5.5 to 19.1% of the total phenotypic variation observed [30].

Overall, although limited in number, these studies point out that drought responses in poplar are complex, as they are controlled by multiple loci. In future works, fine mapping of the identified QTLs may lead to the identification of candidate genes which can be functionally characterized. This would allow the understanding of their contribution to the phenotypes displayed by poplar plants exposed to water-limiting conditions.

## 3. Mining Poplar Transcriptome for Seeking Pathways and Genes Involved in Drought Response

Transcriptomics, based on microarrays or direct sequencing of mRNAs (RNA-Seq) or microRNAs (miRNA-Seq), has been widely applied to investigate the molecular bases of responses to drought in poplar. This approach has allowed the analysis of pathways and genes differentially expressed under specific stress treatments in different poplar species, varieties, or tissues. The results have led to the identification of candidate or marker genes that could be used in breeding programmes [31].

For example, the GeneChip Poplar Genome Array (Affymetrix) was used to compare the transcriptional response of root apices and leaves from the *P. deltoides* × *P. nigra* hybrids Carpaccio and Soligo, which display contrasting tolerance to drought. The results, which were further supported by a meta-analysis of expression data from previously published studies, allowed the identification of genes and processes differentially regulated in roots and leaves during rapid and long-term responses to drought [32]. The same chip was used to mine the response of *P. euphratica*, a poplar species adapted to arid environments, exposed to water deficit for 7 weeks at four different intensities. The analyses revealed variability among different regimes, suggesting that specific regulatory pathways are activated according to the severity of the imposed stress. Candidate marker genes were detected belonging to transcription factor and heat shock protein (HSP) families [33].

While genes and pathways involved in drought responses are largely conserved in plants, in specific tissues and conditions lesser-known molecular patterns can be activated [34]. For instance, in the expression profile of drought-stressed roots of *P. nigra* plants, genes responding to oxidative stress, including some encoding reactive oxygen species (ROS) scavenging enzymes, were downregulated [35]. A significant downregulation of genes involved in protection from oxidative stress was also detected in the transcriptome of *P. trichocarpa* xylem parenchyma cells after the induction of embolism [36]. In this case, the downregulation could have been due to a reduction in the abundance of ROS that may be part of an embolism-sensing molecular mechanism. Furthermore, a transcriptomic study on the response of *P. euphratica* plants exposed to four different intensities of drought stress showed that under moderate drought stomatal closure was inhibited concomitantly to transcriptional remodelling of signal transduction, photoprotection, ROS detoxification, and stress responsive genes. The results provided a list of candidate genes implicated in the inhibition of stomatal closure and in the modulation of the ascorbate–glutathione and ubiquitin–proteasome pathways, setting the basis for understanding the specific adaptation mechanisms evolved in this species [37]. ROS production under drought conditions is not entirely detrimental, as it is part of an important stress-triggered signalling pathway. *P. trichocarpa* plants overexpressing *WRKY75*, a member of a transcription factor family characterized by the conserved amino acid sequence WRKYGQK, displayed higher rates of growth and photosynthesis under drought conditions. *PtrWRKY75* activated the expression of *PHENYLALANINE AMMONIA LYASE 1* (*PAL1*), which resulted in increased accumulation of salicylic acid (SA) and ROS that acted as signals to induce stomatal closure [38].

Microarray technology was applied to mine the transcriptional responses to drought in poplar genotypes that showed different degrees of adaptation to water scarcity [39,40,41,42,43,44,45,46,47,48]. For instance, the transcriptional profiles of three *P. nigra* clones with different behaviour under limiting water conditions were analyzed. The tolerant clone displayed enrichment of genes encoding bark storage proteins and HSPs, while the avoidant and the drought-escape clones activated genes implicated in secondary metabolism, programmed cell death and leaf senescence [39]. Intraspecific variation was also found among six different *P. balsamifera* genotypes exposed to water-withdrawal treatments. The genotypes differed in terms of transcriptional responses and metabolite accumulation, including sugar accumulation, citric acid metabolism, and raffinose family oligosaccharide biosynthesis [40]. In addition, the transcriptional response of leaves from *P. simonii*, a widespread and major pioneer tree species in China, revealed that drought activated genes implicated in phytohormone metabolism, osmoregulation, oxidative stress, carbohydrate metabolism, and amino acid transport [41].

Drought is closely connected with other abiotic stresses, particularly with salt stress and heat stress. Salt stress lowers soil water potential, reducing water availability for root uptake, and can damage roots directly by ion toxicity. High temperatures induce stomatal opening that results in increased transpiration and aggravates water loss [37,42]. A microarray analysis of the response to drought and salt stress in the leaves of a *P. alba* × *P. glandulosa* hybrid allowed the identification of genes that were modulated by both stresses, evidencing crosstalk between the two response patterns [43]. In plants of *P. simonii* exposed to a combination of heat and drought stress, genes modulated by single or combined conditions were identified in roots and leaves. These included genes implicated in RNA regulation, transport, hormone metabolism, and stress responses [43]. Furthermore, high temperature and/or drought activated abscisic acid (ABA) accumulation but repressed the metabolism of auxin and other phytohormones [44]. Similar analysis was applied to leaves of *P. deltoides* × *P. nigra* hybrids exposed to water stress, providing useful resources for comparative studies among *Populus* species [45,46]. Furthermore, the response of *P. deltoides* leaves to single or combined ozone and drought stresses was studied by RNA-Seq. The results indicated that the molecular response to drought was counteracted by elevated ozone. Stomatal closure and the activation of isoprene biosynthesis under ozone and drought were associated to the activation of the ABA and non-mevalonate-dependent pathways, respectively [47]. However, in *P.* × *euramericana*, activation of the mevalonate pathway leading to terpenoid biosynthesis by overexpression of the rate-limiting enzyme hydroxy-3-methylglutaryl coenzyme A reductase (HMGR) was shown to confer improved drought and salinity tolerance, to upregulate the expression of ROS-scavenging enzymes, and to promote root development [48].

In addition to the findings made by mRNA analyses, miRNAs responsive to limiting water availability were identified in *P. euphratica* through high-throughput sequencing. In one study, 197 miRNAs conserved between *P. euphratica* and *P. trichocarpa* were detected, while 58 new miRNAs belonging to 38 families were specific to *P. euphratica*. Degradome sequencing allowed characterization of the target genes of 26 new and 21 conserved miRNAs potentially implicated in the regulation of drought response [49]. A similar analysis was applied to *P. trichocarpa*, in which among the miRNAs showing notable transcription changes upon drought treatment, nine were already known to be involved in drought stress-response, while three were not. From degradome data, 53 and 29 genes were respectively identified as targets of conserved and new miRNAs, and were found to be involved mainly in the regulation of transcription factors, stress responses, and lipid metabolism [50].

## 4. Identification of Genes and Pathways Modulating Stomatal Development and Function

Stomata regulate gas exchanges in plant leaves, allowing CO_2_ uptake for photosynthesis and H_2_O efflux to drive water and solute fluxes from soil to leaves. The regulation of these exchanges is of primary importance in adaptation strategies to different levels of water availability. Genes and pathways controlling stomatal conductance have been identified in model plants and economically important crops, including poplar, and are considered promising targets for breeding programmes and biotechnological applications. These molecular actors fall into two major categories: control of stomatal development and regulation of the stomatal aperture [51].

A comprehensive set of genes controlling stomatal development in poplar was identified by sequence homology with their Arabidopsis counterparts [52]. These include *STOMAGEN*, encoding a signal peptide that acts as a positive regulator of stomatal development, *ERECTA* (*ER*), which encodes leucine rich repeat (LRR) receptor like kinases, *STOMATA DENSITY AND DISTRIBUTION 1* (*SDD1*), encoding a protease that acts as negative regulator of stomatal development, *FAMA*, encoding a basic helix-loop-helix (bHLH) transcription factor, *YODA* (*YDA*) encoding MAP kinase kinase kinase that starts a MAP kinase signalling cascade that negatively regulates stomatal development, and *TOO MANY MOUTHS* (*TMM*), encoding an LRR-receptor-like protein that modulates stomatal patterning [52]. Environmental cues modulate the expression of these regulators. In two genotypes of *P. balsamifera* exposed to drought, newly formed leaves were characterized by reduced stomatal index (the ration between stomatal density and the sum of stomatal density and epidermal cell density). This reduction was correlated mainly with the expression of *STOMAGEN* and *FAMA* [52]. Interestingly, an experiment with isolated upper and basal portions of poplar plants showed that environmental cues (CO_2_ concentration, vapour pressure deficit, shading) sensed by the basal, fully developed leaves affected the stomatal density of the upper, developing leaves [53]. This suggests the involvement of a systemic signal, whose nature, however, remains under investigation. In *P. tremula* × *P. tremuloides*, a fast-travelling signal modulating stomatal conductance was hypothesized to be transmitted by ROS, Ca^2+^, or electric waves [54]. However, other signals may be involved in the longer-term adaptation mechanisms that modify stomatal density.

Regulators of stomatal development have been overexpressed in several poplar species and hybrids (Table 1). From the hybrid poplar clone NE-19, Wang et al. [55] isolated *PdEPF1*, a homologue of Arabidopsis *EPIDERMAL PATTERNING FACTOR 1* which encodes a signal peptide that inhibits the progression towards guard cell differentiation, acting antagonistically to STOMAGEN. *PdEF1* was induced by drought stress and ABA, and *P. tomentosa* plants overexpressing *PdEPF1* displayed reduced stomatal density on the abaxial leaf surface, higher water use efficiency (WUE) and increased drought tolerance [55]. WUE, the amount of biomass assimilated per unit of water used, is a key concept used to evaluate plants’ productive potential under different levels of water availabilty [56]. Interestingly, in well-watered conditions the growth rate and the photosynthetic rate of wild-type and *PdEPF1*-overexpressing plants was similar, indicating that no yield penalty was conferred by the transgene [55]. Based on these results, *PdEPF1* can be regarded as an interesting candidate gene for breeding and biotechnology programmes.

The set of genes isolated from the high-WUE *P. nigra* × (*P.deltoides* × *P.nigra*) hybrid NE-19 included *PdERECTA* and the signal peptide-encoding *PdEPFL6* [57,58,59], both negative regulators of stomata differentiation. Overexpression of *PdERECTA* and *PdEPFL6* also resulted in decreased stomatal density and increased drought tolerance without a significant reduction in biomass accumulation under non-limiting water conditions. However, when the positive regulator of stomatal development *STOMAGEN* was overexpressed in the poplar hybrid clone 84K, the plants displayed increased stomatal density and conductance, higher net photosynthetic rate, and higher vegetative growth [60]. In other cases, the complexity of the regulatory circuits involved in the control of stomatal differentiation makes it difficult to examine fully the effects of their perturbation [61,62]. This is exemplified by the manipulation of *GTL1*, a homologue of Arabidopsis *AtGT-2-like1* (*GTL1*) which encodes for a transcriptional repressor of *SDD1* [61]. In *P. ussuriensis PuGTL1* was found to be targeted and posttranscriptionally silenced by miR172; plants overexpressing miR172 displayed reduced expression of *GTL1*, reduced stomatal density and transpiration, increased WUE, and growth retardation. However, when *GTL1* was repressed directly, plants displayed similarly altered stomatal density and physiological parameters (stomatal conductance, transpiration, photosynthesis) but not the stunted phenotype of miR172-overexpressing plants [62].

Overall, published works suggest that reduction of stomatal density is a promising strategy for breeding or biotechnological programs aimed at obtaining drought resilient poplar genotypes [55,56,57,58,59,60,61,62]. However, field-based trials remain desirable, as some observations made in natural conditions are not fully in agreement with those in confined environments. In fact, several reports have highlighted that increased, rather than decreased, stomatal density in forest trees can be regarded as an evolutionary adaptation to dry environments [63,64,65]. Denser stomata tend to be smaller and faster at closing or opening in response to changing environmental conditions, allowing more efficient gas exchange resulting in reduced risk of hydraulic dysfunction. Furthermore, in a field trial with 18 hybrid clones from different crosses (*P. deltoides* × *P. trichocarpa*, *P. deltoides* × *P. maximowiczii*, *P. deltoides* × *P. nigra*) subjected to deficit irrigation, the degree of drought resistance, expressed as the ability to maintain higher levels of biomass production under water stress, was shown to correlate directly with abaxial stomatal density [66].

Natural variation in stomatal traits in *P. trichocarpa* has been addressed by GWAS using genotypes collected from the species’ natural range in north-western America and Canada. Genotypes originating from northern latitudes or higher altitude areas were characterized by increased adaxial stomatal density, which along with faster growth may compensate for the limitations posed by a shorter growing season [67,68]. GWAS enabled the association of several genes and alleles with stomatal traits, including *PtSPCH1*, a *P. trichocarpa* homologue of Arabidopsis *SPEECHLESS 1* (*SPCH1*) which encodes a bHLH transcriptional activator of stomata differentiation [68]. The identification of *PtSPCH1* alleles associated with adaxial stomatal density paves the way for their possible transfer to other sexually compatible genotypes using a cisgenic approach. Furthermore, a multitrait GWAS approach integrated with co-expression network analysis recently led to the identification of potential regulatory networks underlying three key traits related to yield and responses to abiotic stresses: carbon isotope composition (which was used as an estimate for WUE), stomatal density and leaf area [69]. These studies illustrate the potential of the GWAS approach for mining the genetic variation underlying interesting phenotypic traits.

Stomatal closure under water-stress conditions is mainly induced by ABA perception in guard cells, which is mediated by the PYR1/PYL (pyrabactin resistance 1/PYR1-like)/regulatory component of ABA receptors (RCARs) proteins [70,71]. Overexpression of poplar PYR1/PYL/RCAR receptors in Arabidopsis conferred enhanced ABA sensitivity and drought tolerance to the plants [72,73,74], suggesting that this strategy could be used to obtain drought-resilient poplar genotypes. Accordingly, in a controlled greenhouse environment, transgenic *P. davidiana* × *P. bolleana* poplars overexpressing two ABA receptors (*PtPYRL1* and *PtPYRL5*) from *P. trichocarpa* displayed increased biomass accumulation and reduced oxidative stress under drought conditions, while they did not differ significantly from the wild type under non-limiting water conditions [74]. Despite these promising results, further work is needed since experiments in field conditions and with different poplar genotypes have shown slightly different responses in this case and others. For instance, in a long-term field experiment, *P. tremula* × *P. tremuloides* transgenic lines overexpressing the *RCAR1/PYL9* ABA receptor displayed surprisingly increased biomass accumulation under non-limiting water conditions, but drought tolerance was not improved [75]. Furthermore, overexpression of several components of the ABA signalling pathway, including members of type 2C protein phosphatases (PP2C) gene family, genes encoding ABA-responsive element binding (AREB) transcription factors, and genes encoding basic region/leucine zipper motif (bZIP) transcription factors evidenced that the activation of ABA signalling exerts a repressive action on leaf and shoot growth while promoting root growth, which can result in reduced biomass accumulation in conditions of non-limiting water availability [76].

Other large families of transcription factors well known for their role in ABA signal transduction include V-myb myeloblastosis viral oncogene homolog (MYB) proteins, myelocytomatosis (MYC) proteins, NAM, ATAF1,2, and *C*UC2 (NAC) proteins, and WRKYs [77,78,79]. In poplar, some genes of these families have been characterized. For instance, Arabidopsis and *P. tomentosa* plants overexpressing *PtrMYB94*, a drought- and ABA-induced transcription factor from *P. trichocarpa*, displayed increased ABA accumulation, expression of ABA- and drought-responsive genes, and drought tolerance [77]. In addition, some lesser-known regulators of stomatal function have been characterized, including for instance PdGNC (GATA, nitrate-inducible, carbon-metabolism involved), a GATA transcription factor (a member of a class of proteins characterized by their ability to bind to the DNA sequence “GATA) induced by ABA and dehydration that activates the expression of *PdHXK1*, a hexokinase (HXK)-encoding gene. Increased expression of *HXK1* in guard cells results in the production of NO and H_2_O_2_, that act as signal molecules to induce stomatal closures [78]. Moreover, the *P. trichocarpa* gene PtXERICO, encoding for a RING (really interesting new gene)-H2 zinc finger E3 ubiquitin ligase, was shown to be induced by ABA and to increase drought tolerance by conferring ABA hypersensitivity when overexpressed in Arabidopsis plants [79]. Overexpression of another drought-, ABA-, and salinity-induced E3 ubiquitin ligase-encoding gene, *Plant U-BOX (PUB) 79* (*PalPUB79*) from *P. alba*, conferred enhanced drought tolerance to plants, while the inhibition by RNAi resulted in higher sensitivity. *PalPUB79* was shown to interact with *PalWRKY77*, a negative regulator of ABA signal transduction, mediating its ubiquitin-mediated degradation [80].

## 5. Control of Non-Stomatal Water Loss by Cuticular Waxes

The aerial parts of land plants are protected by the cuticle, an extracellular hydrophobic layer composed of the cutin polymer, which is insoluble in organic solvents, and cuticular waxes that are mainly composed of a mixture of long-chain fatty acids, aldehydes, alkanes, ketones, alcohols and esters. The cuticle constitutes a barrier that protects from excessive water loss, UVB, temperature stress and biotic attacks [81,82]. The modification of the cuticle, particularly of the wax fraction, has been explored as a possible strategy to increase drought tolerance in poplar. By mining transcriptome data from drought-stressed plants and by homology with the Arabidopsis gene *WAX INDUCER1/SHINE1* (*WIN1/SHN1*), a positive regulator of cuticular waxes biosynthesis [83], *PeSHN1*, a member of the APETALA2/ethylene responsive factor (AP2/ERF) transcription factor family, was isolated from the elite *P.* × *euramericana* clone ‘Neva’ [84]. *P. alba* × *P. glandulosa* 84K plants overexpressing *PeSHN1* displayed increased wax accumulation, decreased transpiration and increased photosynthetic activity and WUE under drought conditions. *PeSHN1*-overexpressing plants also displayed altered wax composition, with a shift towards longer-chain fatty acids, aldehydes and alkanes that probably contributed to limiting transpiration [84]. A subsequent study showed that the molecular composition, but not the total amount of waxes, determined the level of cuticular transpiration in different poplar species and hybrids [85,86]. Interestingly, overexpression of *AtSHN1* in Arabidopsis, as well as overexpression of its homologues *TdSHN1* from durum wheat in tobacco and *TaSHN1* from *Triticum aestivum* in wheat, also caused reduction in stomatal density [87,88,89]. It would be interesting to discover whether the poplar plants overexpressing *PeSHN1* also have altered stomatal density. In addition, considering that the clone ‘Neva’ is characterized by high amounts of cuticular waxes, it would be interesting to compare the sequence and the activity of the *PeSHN1* allele used for the transformation with those of its homologues from other poplar genotypes. The identification of superior alleles has been the subject of increasing attention by researchers for their potential use in biotechnological approaches, considered more acceptable to the public such as cisgenesis [90].

## 6. Enhancing Water Uptake and Belowground Traits

As specialized organs for water and nutrient uptake, roots play important roles in plant responses to drought. In poplar, root traits are highly plastic; under moderate water limiting conditions the root system adjusts its growth, which results in several morphological changes and increased ratio of roots to shoots [91,92]. The ability of the root system to access deep or fluctuating water tables is considered a key adaptive trait of poplar species that have evolved in arid regions, such as *P. euphratica* [93,94]. Root traits are promising targets for breeding programs; the ratio between fine root length and total leaf area has been identified as a useful early-stage proxy for drought resilience in selected poplar hybrids [95].

Root-growth dynamics under limiting water availability are controlled by ABA and auxin [96]. Yu et al. [75] characterized several *P. tremula* × *P. tremuloides* transgenic lines overexpressing or downregulated for different genes involved in ABA signalling. Upregulated lines, most notably those overexpressing the transcription factor *AREB3*, displayed increased drought tolerance and biomass reallocation with increased root/shoot ratio under drought conditions, and severely reduced productivity under well-irrigated conditions [75]. In this case, the drought tolerance/yield trade-off may have been due to the use of the strong constitutive promoter 35S, leading to excessive expression levels of the transgenes; therefore, it would be interesting to repeat the experiment using a stress-inducible promoter. The interplay between ABA and auxin signalling in the control of root growth under water limiting conditions has been investigated in transgenic poplars overexpressing or downregulated for *NUCLEAR FACTOR-YB21* (*PdNF-YB21*), a transcription factor involved in drought responses and ABA signalling. *PdNF-YB21*-overexpressing plants displayed increased root growth and drought tolerance, while knock-out mutants obtained by the CRISPR (clustered regularly interspaced short palindromic repeats)–Cas9 (CRISPR-associated protein 9) system, an up-to-date method of targeted mutagenesis, showed the opposite phenotype [97]. *PdNF-YB21* was shown to interact with a second transcription factor, *PdFUSCA3* (*PdFUS3*), which directly activated the transcription of *PdNCED3*, a gene encoding a rate-limiting enzyme of ABA biosynthesis. The resulting increased ABA concentration promoted auxin transport to the root tips, which in turn stimulated root growth [97]. 

Drought-induced morphological modifications such as increased growth and hydrotropism are integrated by functional adaptations. For instance, the transcription factor *PalERF2*, an AP2/ERF transcription factor from *P. alba* var. *pyramidalis*, is upregulated by drought and promotes the expression of Pi transporters that improve Pi uptake under drought-stress conditions [98]. Furthermore, rerouting of gene expression patterns in roots under drought stress affects physiological adaptations at the whole plant level. Under mild drought, *P. tremula* × *P. alba* lines downregulated for the tonoplast sucrose transporter (SUT) *PtaSUT4* displayed reduced shoot growth compared to the wild type. *SUT4*-RNAi plants displayed altered expression patterns that were more significant in root tips for genes encoding LEAs and for ABA-, ethylene (ET)-, and jasmonate (JA)-associated genes, and in stem xylem for aquaporin-encoding genes. These results suggest that the shoot/root sugar gradient mediated by PtaSUT4 affects the response of the whole plant to drought by modulating specific gene-expression patterns in different organs and tissues [99].

Research efforts to elucidate the molecular bases of root responses to drought in poplar have disclosed new functions of genes that were previously characterized in herbaceous model plants. For instance, the basic region/leucine zipper 1-like (*PtabZIP1L*) transcription factor from *P. tremula* × *P. alba* was shown to promote lateral root growth under osmotic and drought stress, in addition to its role in the modulation of flavonoid and proline biosynthesis previously described in Arabidopsis [100]. Furthermore, transcriptional network analyses in *P. tremula* × *P. alba* identified nine superhub genes, two of which, *PtaJAZ3* and *PtaRAP2.6*, are homologs of Arabidopsis *JAZ3* (jasmonate-zim_domain protein 3) and *RAP2.6* (related to Apetala 2.6) genes that are involved in jasmonate and ABA signalling. Functional analysis in transgenic poplars showed that *PtaJAZ3* and *PtaRAP2.6* stimulates root elongation and lateral root proliferation specifically during drought stress. Both superhubs are induced by methyl jasmonate (MeJA), and *PtaJAZ3* induces the transcription of *PtaRAP2.6* [101]. These results increased our knowledge of the complex networks that control root growth in limiting water conditions, highlighting the usefulness of poplar as a model tree; additional works in different tree species would allow researchers to assess the degree of genetic conservation of these networks.

As industrial poplar genotypes are maintained and multiplied clonally by cuttings, the ease of adventitious root development has been the subject of intense research and a goal for selection programmes. The molecular players controlling adventitious root development and those controlling root plasticity under limited water availability are partially overlapping. For instance, WUSCHEL-related homeobox (WOX) transcription factors have been characterized as regulators of cell division and root formation in several plant species, and as determinants of rooting ability in poplar cuttings [102,103]. Recently, it was shown that 84K poplars overexpressing root-specific and drought-induced *PagWOX11/12a* displayed increased root biomass and drought tolerance while opposite phenotypes were displayed by *PagWOX11/12a*-downregulated plants [104]. 

Plants’ belowground environments host a variety of microorganisms that influence the physiological status of co-residing plants under limiting- and non-limiting-water conditions [91,105]. These microorganisms establish different degrees of associations with the plant roots and can promote growth and drought tolerance through a variety of mechanisms including extension of the root absorbing surface, facilitation of ion mobility and uptake, modulation of gene expression patterns and hormonal pathways, stimulation of antioxidant capability, modulation of water transport and stomatal movement [106]. Deep sequencing-based investigations of the poplar root-associated microbiome have evidenced variations between plant niches, geographical regions and seasons [107,108].

Root-associated microorganisms have been shown to modulate the expression of genes potentially involved in drought resistance. Plants of *P. tremula × P. tremuloides* inoculated with an ectomycorrhizal fungus and exposed to drought stress displayed increased expression of two aquaporin genes, suggesting that these genes may contribute to water uptake and transport under stress conditions [109]. Poplar establishes both ecto- and endomycorrhizal symbiosis, but ectomycorrhizal fungi have been reported to be more effective in increasing root hydraulic conductivity of *P. balsamifera* plants [91,110].

The composition of the poplar root-associated microbiome is modified by the characteristics of the soil and, interestingly, by the genotype of the host plant [111]. Furthermore, it has been shown that the composition of the poplar root-associated microbial community changes in responses to acute and prolonged drought episodes [112,113,114]. It is noteworthy that the root-associated microbiome of *P. trichocarpa* drought-adapted genotypes displays greater changes then that of drought-sensitive genotypes. Thus, the root environment of drought-tolerant genotypes exposed to drought stress is enriched in microorganisms, particularly bacteria and ectomycorrhizal fungi, that can play beneficial roles in stress conditions [114]. Future work may clarify the factors (e.g., metabolites and root exudates) that shape the root microbiomes of drought-tolerant poplar genotypes. Interestingly, poplar plants inoculated with a set of microorganisms including bacteria and fungi isolated from the rhizosphere of drought-stressed plants displayed increased root elongation, plant height and relative water content, compared with non-inoculated plants, indicative of growth-promoting and drought-protective action [113].

## 7. Epigenetic Regulation of Drought Responses

A rapidly increasing series of studies has addressed the epigenetic mechanisms underlying the responses to drought and related abiotic stresses in woody plants [115]. Epigenetic mechanisms involve histone and reversible DNA modifications, independent of variations in DNA sequence, that alter chromatin structure with consequences for gene expression [116]. These mechanisms are associated with the phenomena of stress memory and priming that allow plants to display a stronger, faster, or more sustained response to a recurrent stress condition when they have previously been exposed to it [117,118,119].

One of the first hints of the adaptive roles of epigenetic mechanisms in forest trees came from observations made in provenance trials with Norway spruce, showing that plants which developed from seeds originating from areas with contrasting ecological conditions displayed phenological dynamics that correlated with their origins [120,121]. A similar effect was reported in poplar with agamic reproductive material. In poplar hybrids propagated by cuttings, it was observed that physiological and transcriptomic responses in the leaves of plants exposed to drought stress were dependent on the geographically and climatically divergent sites where the plants grew from which the cuttings were collected [122,123]. Differences in transcriptome responses were paralleled by differences in genome-wide DNA methylation patterns [122]. Previously, it was reported that global DNA methylation rates in the shoot apexes of six *P. deltoides* × *P. nigra* hybrids were differentially affected by water stress and, correlated directly with yield in non-limiting water conditions [124]. Le Gac et al. [125] confirmed this result and showed, by contrast, that in drought-stress conditions the global methylation status of the dormant shoot apex was negatively correlated with shoot biomass. In their study, differentially methylated regions were detected six months after the application of the stress treatment and were mostly located in gene bodies and promoters. Furthermore, genes of the ET, JA and SA pathways known to be involved in drought responses were differentially methylated and differentially expressed in the transcriptome of shoot apical meristems of poplar plants during drought and recovery treatments [126]. JA- and SA-responsive genes were activated under drought stress and repressed after rewatering. Transcriptional repression after rewatering was associated with gene-body hypomethylation [126]. Decreases in global DNA methylation rate in response to drought were also observed in the shoot apexes of field-grown *P. nigra* plants originating from different natural populations [127]. However, the correlation between DNA methylation and transcription is complex, and gene-body methylation in *P. trichocarpa* has been reported both to promote and repress transcription [128,129].

Interestingly, homologues of genes involved in ABA signalling, including two genes encoding PP2C phosphatases, two encoding *HOMEOBOX7* (*HB7*)-related transcription factors, and two encoding LEAs, showed expression patterns clearly shaped by stress memory in different tissues and organs of *P.* × *canescens* plants exposed to cycles of water stress and recovery [130]. This suggests that ABA signalling, the central response pathway activated under drought stress, which also controls key yield-related traits including biomass allocation between leaves and roots in response to drought [75], may be regulated epigenetically. However, further experiments examining the underlying molecular signatures are required to confirm the involvement of DNA methylation and histone modifications.

Recently, the epigenetic bases of drought responses in poplar plants have been addressed by modifying the expression of *DECREASED IN DNA METHYLATION1* (*DDM1*), which encodes a chromatin remodelling factor that participates in cytosine methylation [131]. Under moderate water-stress conditions, the growth rate of *P. tremula* × *P. alba* plants downregulated for *DDM1* was substantially unaffected, while the height and diameter growth rates of WT were reduced by 25% and 39%, respectively [131]. This drought-tolerant phenotype was associated with increased accumulation of SA and reduced accumulation of cytokinins in the shoot apex. Intriguingly, *DDM1*-downregulated plants also displayed reduced susceptibility to cavitation, which was not associated with morphological variations in the xylem vessels, hinting at a possible epigenetic regulation of active embolism-repair mechanisms.

## 8. Embolism Formation and Repair Mechanisms

Long-distance water transport in vascular plants occurs in the xylem, a network of non-living cells connecting roots to leaves. In certain conditions, such as drought and/or high evaporative demand, the water column within the lumen of the xylem vessels can be subjected to high tension, resulting in embolism formation [132]. Embolisms are caused by the entry and expansion of air bubbles in the xylem [133,134,135], and their presence reduces the capacity of the stem to transport water, and induces plant desiccation, tissue damage and decline of plant productivity [133,136]. High levels of gas-filled conduits (embolized vessels) can lead to hydraulic failure in the xylem, which is the major driver of tree mortality [137]. Embolism formation is a physical phenomenon related to the degree of tension in the water column, water chemical properties, surface tension in the xylem sap, and the anatomical and physicochemical properties of the wood such as diameter, length and connectivity of conduits, density, and pit characteristics [138,139,140,141], as well as to a history of previous embolism activity [142]. It has been suggested that xylem vulnerability to embolism in *P. tremula* × *P. alba* also relies on pit properties such as pit area [143]. 

Vulnerability to embolism is species-specific and tissue-specific, with some plants showing high resistance to hydraulic dysfunction, and others losing hydraulic conductivity at relatively high xylem water potential. P50 is the most widely used physiological parameter to describe vulnerability to embolism formation and predict the drought tolerance of tree species [144,145]. It corresponds to the value of water potential at which 50% of hydraulic conductivity is lost; the greater the negative P50 value, the higher is the plant’s tolerance to stress.

Among plant species, poplar is considered one of the temperate woody plants most vulnerable to drought-induced embolism. A meta-analysis reported a mean P50 close to −1.44 MPa for 10 analysed species of poplar [146], accounting for one third of the total number of poplar species [147]. Data were collected from potted poplars grown under controlled conditions and from trees grown in the field, and P50 values showed significant variation among species, probably due to species-specific ecological range. Among the examined species, *P. tremuloides* and *P. euphratica* appear to be the most resistant and the most vulnerable, respectively, to drought-induced embolism, with a mean P50 of −2.13 MPa for *P. tremuloides* and of −0.70 Mpa for *P. euphratica* [147,148]. As well as species, poplar hybrids also revealed elevated vulnerability to embolism formation; among the12 different hybrids analysed, a mean P50 of −1.55 MPa was reported, although high variation was detected. *P. tremula* × *P. alba*, *P. trichocarpa* × *P. balsamifera* and *P. deltoides* × *P. nigra* represented the three most resistant hybrids, thus confirming the resistance of their respective parental species [147]. 

Different vulnerability to stress in poplars has been also reported within individual trees: older vessels and the basal region of the main stem are more susceptible to embolisms compared with newer vessels and he apical region of the stem, respectively [148,149]. Different environmental conditions can affect hydraulic functionality in poplar; in *P. nigra* prolonged shading increased xylem vulnerability to embolism, probably due to lower sugar content accumulated in stem tissues [150]; in the same species, sugars derived from photosynthesis in woody tissue reduced xylem vulnerability to drought [151].

Plants have evolved several strategies to regain or mitigate the loss of transport capacity in the xylem, including shading leaves, rebuilding new functional xylem and generating root pressure [142,152,153]. It has been observed that even after severe water stress, *P. tremula* and *P. tremuloides* plants despite exhibiting complete leaf and stem desiccation were able to resprout after drought was alleviated [154]. Moreover, *P. tremuloides* trees grown in natural stands and characterized by impaired hydraulic conductance were able to recover their hydraulic functionality during the growing season [155]. However, all these processes are either very slow or require the presence of positive pressure in the tree.

There is mounting evidence that some tree species can recover from embolism in the short term (hours to several days), even when most of the water in the xylem is under low pressure [156,157,158]. Since the process of embolism recovery requires that empty conduits be filled with water against existing energy gradients, this cannot happen spontaneously and needs some physiological activity to drive water flow into the embolized vessel [159,160].

Recently, for poplar, a detailed mechanistic model of xylem hydraulic recovery from embolism has been proposed [161,162]. The model suggests that the living parenchyma cells (or vessel-associated cells, VACs) adjacent to the xylem vessels are directly involved in the restoration of xylem, (i) generating an energy gradient that allows water to flow into empty vessels and (ii) supplying water for recovery. During water stress, the content of soluble sugars (mostly sucrose and maltose) in parenchyma cells increased as result of starch degradation and the need to lower cell osmotic potential in the xylem. The drop in starch concentration coincided with a transcriptional increase in beta and alpha amylase-encoding genes [163,164]. The soluble sugars are actively pumped from living cells to the xylem apoplast via proton-coupled sucrose transporters or via passive efflux through the membrane, where they accumulate as they cannot be carried away with the transpiration stream. In poplar, the efflux of sugars is induced by low apoplastic pH, a condition that could also be promoted by the activity of H^+^-ATPase proton pumps [165]. The new acidic apoplastic environment promotes the activity of acidic invertases, enzymes that split sucrose into glucose and fructose and thus accumulate monosaccharides in the apoplast. Therefore, the lower concentration of extracellular sucrose and the presence of a sucrose gradient between living parenchyma cells and the apoplast promotes further sucrose efflux from parenchyma cells [163]. In response to drought, a reduction of pH in the xylem sap of *P. tremula* × *P. alba* has been measured, and has been observed directly in vivo in stem tissues [161]. These physiological activities are closely coupled to the upregulation of genes involved in maltose and sucrose transport and the encoding of acidic invertases [163] and metal ion transporters [164]. Indeed, lower apoplastic pH could also promote the activity of metal ion transporters providing additional osmolytes needed for embolism recovery. A higher concentration of ions has been reported in the xylem sap of *P. nigra* [165].

The solutes accumulated in the apoplast generate an osmotic gradient that triggers the entry of water into the embolized conduits, allowing for recovery. Water entry is facilitated by aquaporins, plasma-membrane water channels that are upregulated in response to embolism [166,167,168]. In particular, an analysis of the temporal dynamics of expression of poplar PIP1 and PIP2 transcriptional profiles revealed a general strong overexpression of the PIP1 subfamily when water stress occurred [38]. A genome-wide analysis of *P. trichocarpa* stems responding to embolism formation confirmed the specificity of aquaporin-expression patterns [36]. In the same poplar species, the role of water channel proteins in facilitating the recovery of hydraulic conductance in leaves after water stress has been further demonstrated [169]. The suppression of poplar PIP1 expression significantly lowered refilling activity, resulting in an apparent increase in vulnerability to embolism formation in transgenic poplars, thus confirming the crucial role of aquaporins in promoting recovery from stress [170]. A positive correlation between aquaporin expression and differences in the loss of conductivity along the stem of *P. alba* × *P. glandulosa* was found and related to the organs’ ability to repair embolism [171].

## 9. Drought and Wood Quality

Certain physiological, biochemical, and morphological changes deployed by trees in response to drought relate to woody tissues and involve a reorganization of the xylem architecture. Similar to the adaptive morphological adjustment of roots and leaves illustrated previously, wood modifications in poplar are thought to be regulated primarily by ABA, which is the most abundant hormone in the xylem sap [172].

ABA treatments and drought stress cause similar effects in poplar stems. Popko et al. [173] reported that ABA accumulation modulates wood formation leading to an increase in vessel numbers and a decrease in their cross-sectional area, a trait that affects both water conduction and cavitation vulnerability. Several regulators have been characterized that link drought responses to wood modifications. Xu et al. [174] identified the transcription factor *PtoMYB170* from *P. tomentosa* as a molecular determinant of lignin deposition and drought tolerance, indicating that these processes can be co-ordinately regulated. *PtoMYB170* is expressed in leaves and xylem tissue, and its overexpression in transgenic poplars resulted in thickened secondary walls in the xylem compared with controls. GUS reporter expression driven by the *PtoMYB170* promoter in Arabidopsis localized to guard cells, and Arabidopsis plants ectopically expressing *PtoMYB170* displayed increased drought tolerance. Furthermore, Wang et al. [175] showed that the overexpression of *PtaERF194* in poplar hybrids (*P. tremula* × *P. alba*) resulted in growth inhibition associated with an increase in the number of stem xylem vessels and a reduction in their lumen area. Under drought conditions, *PtaERF194*-overexpressing plants also displayed lower stomatal conductance and transpiration, higher leaf water potential, and the transcriptional activation of a set of genes involved in drought resistance, including some known to be controlled by ABA [175]. However, since ERF transcription factors are involved in ABA-dependent and ABA-independent signalling, both pathways may have contributed to the phenotype [176].

The effects of drought-induced alterations on wood quality are still poorly understood. Cocozza et al. [177] evaluated these effects using the commercial poplar clones ‘Dvina’ and ‘I-214’. Under reduced water availability, the clone ‘Dvina’ showed higher Klason lignin content and wood density than ‘I-214’; the clone ‘Dvina’ also showed faster recovery after drought stress, although the wood displayed lower technological properties than ‘I-214’. The increase in fiber numbers and wall thickness induced by drought stress result in higher wood density, which affected the wood’s properties. Increased numbers of vessels associated with higher hydraulic conductivity reportedly caused higher shrinkage leading to the disruption and collapse of the wood’s structure during steam treatment [178]. Interestingly, the extent of drought-induced modifications in the properties of wood varies according to the time in the growing season at which the stress is exerted; *P. nigra* × *P. maximowiczii* plants exposed to drought stress in early summer displayed a significant reduction of fiber length, while with drought stress in late summer the effect was minimized [179].

## 10. Water Stress Versus Biotic Adversities

Water stress in poplar is not only damaging per se, but is also connected with a variety of biotic adversities, sometimes inhibiting and more often enhancing them [180]. Primary pathogens and weak pathogens can be differentiated with respect to their response to water stress. Foliar primary pathogens usually show a decrease in their incidence, due either to the non-suitability of drought conditions for their propagule dissemination and germination, to the reduced patency of stressed leaf tissues for colonization, or to their substantial extraneity to the host microbiota and consequently inability to take advantage of its stress-induced imbalance. For example, *Drepanopeziza brunnea* (Ellis & Everh.) Rossman & W.C. Allen, the Marssonina leaf spot agent, requires rain or at least dew to disperse conidia aggregated in acervula on the leaf surface and to dilute germination-inhibiting compounds [181]. Even if the fungus can benefit from the high temperatures usually associated with drought, low precipitation represents the limiting factor [182]. Although less dependent on a water film on the leaf surface for conidial dispersion, *Melampsora* spp., pathogenic agents of poplar foliar rusts, are likewise not fostered by long drought periods: on poplars, urediniospores, the main conidial stage responsible for multiple generations, only germinate under saturating humidity. Moreover, urediniospore germ tubes penetrate into leaf tissue by stomata, thus they are indirectly penalized by water stress as it induces progressive closing of the stomatal pores [183,184].

Although primary pathogens can be disadvantaged by water-stress-induced host responses, water stress can predispose the host to attacks from a wide group of weak pathogens that may prove even more damaging to the quality of wood. The concept of host predisposition to disease has evolved from Yarwood’s pioneering definition of ”an internal degree of susceptibility resulting from external causes” [185], to more recent views focused on the pathogen inserted into a host environment, an abiotic external environment and a microbial environment, wherein a pathogen “may cause disease individually but may be more or less efficient depending on the presence of other microbes priming host defences or debilitating host physiology” [186]. In other words, there is a type of endophytic latent pathogen for which it is convenient to speak not only of inoculum potential and inoculum pressure, but also of a threshold over which they are able to pass from the latency phase to the actively pathogenic: “pathogenicity is a consequence of fine-tuned interactions between host, environment, and other organisms” (microbiome) [187]. Water stress is perhaps the main factor, and has been one of the most studied, able to drive a latent pathogen and host-associated microbiota (on the whole defined as pathobiome [188]) to the full expression of disease. Some fungal parasites can be symptomatic even in absence of stress, although less aggressively than in water-stressed trees, while others require a stressed host to start detectable attacks, such as *Cytospora chrysosperma* (Pers.) Fr. on quaking aspen [189] and many other poplar species.

Several factors, either structural, biochemical or biophysical, can lead a water-stressed tree to be more appetible or disadvantaged with respect to a weak or latent pathogen [190]:

- among biochemical changes, a decrease in starch level and a concomitant increase in glucose and fructose levels in various bark tissues; e.g., increased glucose content can enhance attacks by *Armillaria mellea* (Vahl) P. Kumm., a polyphagous root rot agent [191]; similarly, increased concentrations of amino acids can stimulate hyphal growth of *Entoleuca mammata* (Wahlenb.) J.D. Rogers & Y.M. Ju, the *Hypoxylon* canker agent in quaking aspen [192];
- among metabolic changes, the synthesis of antifungal compounds and phytoalexins can be inhibited by water stress, e.g., catechol, salicortin and salicin in quaking aspen versus *E. mammata* [193];
- among anatomopathological features, water stress may delay the formation of necrophylactic periderm, a barrier tissue mitigating colonization by pathogens;
- among biophysical changes, xylem embolisms associated with low water potential may provide preferential routes for the spread of internal pathogens [194].

In any case, drought is not always the main stressor and the weak pathogen the secondary: the former and latter may interact simultaneously, and their combined occurrence bring the tree host to exhaustion, according to the concept of tree decline developed by Manion [195] when studying the quaking aspen/Hypoxylon canker pathosystem.

Regarding insect pests, we can record *Phloeomyzus passerinii* (Signoret), the woolly poplar aphid, among the few reported as being impaired by drought or water stress: its optimal development occurs at moderate temperature (20–25 °C) and air humidity of more than 70%, under the shady microclimate provided by trees near maturity [196]. However, in addition to the reduced suitability of a dryer microclimate after water stress (also considering the associated phylloptosis), more intimate relationships between aphid and host tree are involved. Assuming, according to Cornelissen [197], that sap-feeding insects are more abundant and show increased fitness when feeding on more vigorously growing plants (the “plant vigour hypothesis”), the reduced vigour of trees exposed to drought is per se sufficient to explain aphids’ reduced fitness and, consequently, abundance. In addition, a higher concentration of defensive compounds in stressed trees is also involved, leading to decreased tissue palatability and feeding deterrence [198]. However, with particular reference to the woolly poplar aphid, a further factor may interfere. *P. passerinii* is not merely a sucking insect but, most importantly in poplar susceptible genotypes, is also a gall-inducer, causing damage to trees by the formation of pseudogalls characterized by anomalous hypertrophic and lignified tissues in the cortical parenchyma that hamper sap flow. Increasing intensities of water stress, from mild to sharp, induce proportionally smaller galls and consequent lower support to aphid generations in susceptible poplar clones, whereas in resistant clones—in which pseudogall formation is aborted—moderate water stress may partially revitalize aphid performance due to the higher nutritional value of host tissues [199]. Thus, new plantations of woolly-aphid-resistant clones should be adequately irrigated to avoid possible unexpected, although mild, aphid incidence caused by these dynamics.

It is accepted that moderate drought induces a different allocation of carbon and nitrogen resources so that an increase of defence compounds occurs, whereas severe drought means that the synthesis of these compounds is not further sustained and polyphagous insects may thus be favoured [200]. However, different feeding guilds (suckers, borers, miners, etc.) may show different responses to host water stress. For example, the leaf chewer *Chrysomela populi* L. showed decreased survival and feeding in the presence of experimentally induced water stress, both mild and severe, in hybrid poplars [201]; on the other hand, water-stressed trees may synthetize attractive chemicals,—such as ethanol, monoterpenes and others—and the lower water potential in leaves and stems may be associated with reduced resistance to pest attacks [202].

Finally, high temperatures, often simultaneous with drought occurrence, may enhance insect voltinism independently from the altered conditions of target plants. However, this is not always true. It was found that the larvae of *Popillia japonica* (Newman), the Japanese beetle whose adults frequently feed on poplar leaves, were negatively affected in their survival by high soil temperature and very low soil humidity [203]. Thus, the effect of a drought episode on the occurrence of certain insect pests on poplars must be evaluated on case-to-case basis considering all interacting factors.

## 11. Phenotyping Drought Tolerance Traits

The phenotypic evaluation of poplars has been conducted starting in nurseries up to plantations and replicated in different environments. The evaluated characteristics include rooting attitude, growth habit (trunk straightness, branching, apical dominance), resistance to biotic and abiotic stresses, growth rate, and physical characteristics of the wood such as basal density, colour and hue. For phenotyping the effects of drought stress, characters as growth reduction and changes in metabolic activities can be measured [204]. Several technologies are under investigation to assess key tree allometries such as stem diameter and root extension. Traditional and destructive measurements are limited by time and cost. Recently, remote sensing techniques have attracted increasing interest for their ability to extract information in a short time from large populations.

For measure of tree diameter (diameter at breast height, DBH), different dendrometers have been used based on direct measures, involving physical contact with the stem using callipers or tape, or indirect measurement with laser callipers or smartphones [205]. Generally, indirect measurements from remote dendrometers tend to underestimate the mean tree DBH, but no significant differences are found when comparing smartphone measurements to laser calliper measurements. Direct automatic point dendrometers have been used for surveys of trees’ reactions to environmental changes [206]. These tools provide daily patterns of variations in stem radius and maximum daily shrinkage, parameters that are closely related to the plant’s response to water deficit [207]. One of the remote sensing methods under testing in poplar breeding stands is terrestrial laser scanning (TLS), a reliable method to obtain non-destructive measures of volume allometries with high precision and low cost. This technique can measure both crown volume and tree position and can be used to test optimal spacing requirements in innovative schemes such as mixed or polycyclic plantations. The spatially explicit nature of TLS measurements allows better integration with different remote sensing methods, which can be used in combination, enabling multiscale assessment with different levels of detail [208,209,210].

In poplar breeding programmes, field measurements are beginning to be integrated with standardized high-throughput field phenotyping (HTFP). Low-elevation unmanned aerial vehicles (UAVs) equipped with cameras and remote sensors allow the detection of high-resolution spectral responses. Thermal infrared equipped UAVs have been used to evaluate the response to drought of large breeding *P. nigra* populations, taking advantage of the tight correlation between leaf temperature and stomatal conductance [211]. Multi-platform multi-sensor observations, including Sentinel-2 satellite images, thermal infrared and RGB images, tree measurements and climate data integrated through a machine-learning sharpening technique have been applied to evaluate *P. nigra* progeny under drought vs. well-watered conditions [212]. Combining different technologies can improve the reliability and resolution of the results. Spectral images from satellites are very useful for rapid evaluations of large areas, allowing researchers to collect observations on typology of vegetation cover and the health conditions of trees through direct and indirect measures of temperature, evapotranspiration, and other factors. However, integration with proximal sensing data is still necessary to provide adequate resolution and the flexibility to collect precise phenotypic data at multiple timepoints (for example at several timepoints in the same day). Since ground-level surveys are time-consuming, require numerous personnel and are not always possible (for example on steep slopes or under dense vegetation canopy), UAVs equipped with cameras and sensors can be used to bridge the gap between time-consuming ground-based measurements and satellite/airborne observations [210]. With multispectral cameras, these vehicles can acquire multiple data in a single survey. The disadvantages of UAV application are still linked to battery duration requiring multiple recharges during the day, and to the handling of massive image data requiring dedicated software.

## 12. Conclusion and Perspectives

Research on drought responses in poplar has thus far provided a remarkable amount of published data that has significantly enlarged our knowledge of the physiological and molecular mechanisms involved. Studies conducted on a range of genotypes displaying different degrees of adaptation to water scarcity have allowed researchers to understand that the basic response pathways elucidated in herbaceous model plants, such as Arabidopsis, are largely conserved in poplar. This fact emerges clearly from transcriptomic surveys reporting gene expression patterns in plants exposed to drought stress, from the identification and characterization of genes controlling stomata differentiation, and from molecular data on ABA-mediated control of stomatal movement.

In addition, however, research efforts have shed light on processes and mechanisms that are specific for woody plants. Transcriptomic analysis applied to drought-adapted poplar species as *P. euphratica* and *P. simonii* has disclosed finely regulated responses to different levels of drought stress and to combined drought and heat stress, and has led to the identification of differentially expressed miRNAs that were not previously associated with drought. Novel molecular actors and mechanisms have been discovered in developmental and response pathways involved in water relations such as, for instance, the role of miR172 in stomata differentiation. In other cases, an ecological function has been assigned to previously characterized genes. Thus, in the stomata differentiation pathway, *PtSPCH1* was identified by GWAS as a key factor correlated with the distribution range of *P. trichocarpa*, and allelic variation of *PtSPCH1* was correlated with adaptation to different environmental conditions. Studies on the role of ABA signalling in biomass trade-off and resource reallocation between roots and canopy have enriched our knowledge of long-term responses to drought. These responses have been linked with phenotypic plasticity, molecular memory, and epigenetic modifications, which are emerging as important processes in the stress biology of trees. Finally, intensive research into the physiological and molecular mechanisms involved in xylem embolism has allowed to the construction of models for embolism repair. 

These examples show how physiological and molecular information from poplar research can be used to unveil novel mechanisms of adaptation to drought stress. The large genetic variation among and within the poplar species used for the experiments suggests that the results may have some general applicability. Thus, future studies may be aimed at assessing to what extent these mechanisms are conserved among forest trees and evaluating their roles in shaping forests’ dynamics under changing environmental conditions. Results of research into poplar responses to drought also provides a guide for breeding programs aimed at obtaining drought-resilient clones. The identification of useful genes and, particularly, of superior alleles of these genes, pave the way for their introgression in the genetic backgrounds of valuable commercial genotypes (Table 1). 

**Table 1:** Drought-responsive genes overexpressed or downregulated in transgenic poplars ^1^.

Gene	Species	Encoded Product	Note	Reference
*PtrWRKY75*	*P. tremula*	WRKY transcription factor	Overexpressing plants display increased salicylic acid and ROS accumulation, higher photosynthetic rate, and increased growth under drought conditions.	[38]
*PtHMGR*	*P.* × *euramericana*	Hydroxy-3-methylglutaryl coenzyme A reductase	Overexpression improves drought and salinity tolerance by promoting root development and the expression of genes coding for ROS-scavenging enzymes.	[48]
*PdEPF1*	*P. deltoides*	Signaling peptide	Overexpression induces lower stomatal density and transpiration, increased WUE, increased drought tolerance.	[55]
*PdERECTA*	*P. nigra* × *(P. deltoides × P. nigra)*	Receptor-like kinase LRR-RLK	Overexpressing plants have reduced stomatal density and display increased drought tolerance and WUE under drought conditions.	[57,58]
*PdEPFL6*	*P. nigra* × *(P. deltoides × P. nigra)*	Signaling peptide	Overexpression induces lower stomatal density and transpiration, increased WUE, increased drought tolerance.	[59]
*STOMAGEN*	*P. alba* × *P. glandulosa*	Signaling peptide	Overexpressing plants display increased stomatal density, higher net photosynthetic rate, and higher vegetative growth.	[60]
*PtaGTL1*	*P. tremula* × *P. alba*	Ca^2+^/Calmodulin-binding transcription factor	Overexpressing plants display reduced stomatal density and transpiration, increased WUE, growth retardation.	[61]
*Pu-miR172d*	*P. ussuriensis*	MicroRNA	Overexpressing plants display reduced expression of GTL1, reduced stomatal density, increased WUE, and growth retardation.	[62]
*PePYL6*; *PePYL9*	*P. euphratica*	PYR/PYL/RCAR ABA receptors	Overexpression in Arabidopsis confers increased ABA sensitivity, increased WUE, and drought tolerance.	[69]
*PtPYRL1*	*P. trichocarpa*	PYR/PYL/RCAR ABA receptor	Overexpression in transgenic poplars improves ABA sensitivity and drought-stress tolerance.	[74]
*PtPYRL5*	*P. trichocarpa*	PYR/PYL/RCAR ABA receptor	Overexpression in transgenic poplars improves ABA sensitivity and drought-stress tolerance.	[74]
*RCAR1/PYL9*	*P. tremula* × *P. tremuloides*	PYR/PYL/RCAR ABA receptor	Overexpressing plants display increased biomass accumulation under non-limiting water condition.	[75]
*AREB3*	*P. tremula* × *P. tremuloides*	ABA-Responsive Element Binding protein	Overexpressing plants display increased drought tolerance, biomass re-allocation favouring roots in drought conditions, severely reduced productivity under well-watered conditions.	[75]
*FDL1*, *FDL2*	*P. tremula* × *P. tremuloides*	bZIP transcription factors	Overexpressing poplar lines show reduced root growth and increased drought sensitivity; RNAi downregulated lines show higher biomass allocation to roots under drought conditions.	[75]
*PtrMYB94*	*P. trichocarpa*	MYB transcription factor	Overexpression increases the expression of ABA- and drought-responsive genes and improves drought tolerance.	[77]
*PdGNC*	*P. nigra* × (*P. deltoides* × *P. nigra*)	GATA transcription factor	Overexpression in poplar plants results in increased hexokinase expression in guard cells, production of NO and H_2_O_2_, stomatal closure and increased drought resistance. CRISPR/Cas9-mediated mutagenesis results in increased stomatal aperture.	[78]
*PtXERICO*	*P. trichocarpa*	RING-H2 zinc finger E3 ubiquitin ligase	Overexpression in Arabidopsis confers ABA hypersensitivity and drought tolerance.	[79]
*PalPUB79*	*P. alba*	E3 ubiquitin ligase	Overexpressing plants display increased drought tolerance and upregulated ABA signaling due to ubiquitin-mediated degradation of PalWRKY77, a negative regulator of ABA signal transduction; RNAi lines display the opposite phenotype.	[80]
*PeSHN1*	*P. alba* × *P. glandulosa*	AP2/ERF transcription factor	Overexpressing plants display increased cuticular wax accumulation, decreased transpiration, higher photosynthetic activity and WUE in drought conditions.	[84]
*PdNF-YB21*	*P. deltoides*	Nuclear factor-Y transcription factor	Overexpression results in increased ABA concentration, promotion of auxin transport to root tips, increased root growth and drought tolerance; CRISPR/Cas9-mediated mutants display opposite phenotype.	[97]
*PtaSUT4*	*P. tremula* × *P. alba*	Sucrose transporter	Under mild drought conditions RNAi downregulated lines display reduced shoot growth compared to wild type and altered gene expression patterns in root tips and stem xylem.	[99]
*PtabZIP1*	*P. tremula* × *P. alba*	bZIP transcription factor	Overexpression in poplar plants results in increased lateral root development under drought and osmotic stress conditions.	[100]
*PtaJAZ3*	*P. tremula* × *P. alba*	Jasmonate ZIM-domain protein	Overexpressing plants display increased lateral root proliferation under drought stress.	[101]
*PtaRAP2.6*	*P. tremula* × *P. alba*	Related to *Apetala 2.6* transcription factor	Overexpressing plants display increased lateral root proliferation under drought stress.	[101]
*PagWOX11/12a*	*P. alba* × *P. glandulosa*	*WUSCHEL*-related homeobox (WOX) transcription factor	Overexpression results in increased root biomass and drought tolerance; downregulation results in the opposite phenotype.	[104]
*PtDDM1*	*P. tremula* × *P. alba*	SWI/SNF chromatin remodelling factor	Silenced lines display DNA hypomethylation, increased drought tolerance, increased accumulation of salicylic acid and reduced accumulation of cytokinins in the shoot apex.	[127]
*PtaPIP1.1*	*P. alba* × *P. tremula*	Acquaporin	Downregulated poplars show increased vulnerability to embolism and reduced stomatal control of transpiration.	[169]
*PtoMYB170*	*P. tomentosa*	MYB transcription factor	Overexpression results in stronger lignification, thickened secondary wall in xylem and enhanced stomatal closure; downregulation weaken lignin deposition.	[173]
*PtaERF194*	*P. tremula* × *P. alba*	AP2/ERF transcription factor	Overexpression results in increased number of stem xylem vessels and growth inhibition. Under drought conditions overexpressing plants display lower stomatal conductance and transcriptional activation of genes involved in drought resistance.	[174]

^1^ Genes ordered by reference number.

## Data Availability

Not applicable.
